# Review and comparison of quality standards, guidelines and regulations for laboratories

**DOI:** 10.4102/ajlm.v1i1.3

**Published:** 2011-12-13

**Authors:** Tjeerd A.M. Datema, Linda Oskam, Paul R. Klatser

**Affiliations:** 1 KIT Biomedical Research, Royal Tropical Institute, Amsterdam, The Netherlands; 2 Athena Institute, VU University Amsterdam, Amsterdam, The Netherlands; 3 Amsterdam Institute for Global Health and Development, Academic Medical Centre of the University of Amsterdam, Amsterdam, The Netherlands

## Abstract

**Background:**

The variety and number of laboratory quality standards, guidelines and regulations (hereafter: quality documents) makes it difficult to choose the most suitable one for establishing and maintaining a laboratory quality management system.

**Objectives:**

There is a need to compare the characteristics, suitability and applicability of quality documents in view of the increasing efforts to introduce quality management in laboratories, especially in clinical diagnostic laboratories in low income and middle income countries. This may provide valuable insights for policy makers developing national laboratory policies, and for laboratory managers and quality officers in choosing the most appropriate quality document for upgrading their laboratories.

**Method:**

We reviewed the history of quality document development and then selected a subset based on their current use. We analysed these documents following a framework for comparison of quality documents that was adapted from the Clinical Laboratory Standards Institute guideline GP26 *Quality management system model for clinical laboratory services*.

**Results:**

Differences were identified between national and international, and non-clinical and clinical quality documents. The most salient findings were the absence of provisions on occurrence management and customer service in almost all non-clinical quality documents, a low number of safety requirements aimed at protecting laboratory personnel in international quality documents and no requirements regarding ethical behaviour in almost all quality documents.

**Conclusion:**

Each laboratory needs to investigate whether national regulatory standards are present. These are preferred as they most closely suit the needs of laboratories in the country. A laboratory should always use both a standard and a guideline: a standard sums up the requirements to a quality management system, a guideline describes how quality management can be integrated in the laboratory processes.

## Introduction

After the development of the plan-do-check-act cycle by Deming (based on the work of Shewart) in the 1920s,^1,2^ the principles of quality management (QM) spread and became especially important in private industry. QM has many advantages, including protecting the product quality, customer safety and the company’s reputation. Requirements for regulation of processes are recorded in documents called ‘standards’. National and international quality standards were developed by several organisations. Simultaneously, governments forced the introduction of QM by means of laws and regulatory standards.

In laboratory practice the necessity of QM also became apparent, although later than in industry. Laboratory quality standards first focused only on testing laboratories and calibration laboratories, with the first international standard (ISO Guide 25) launched in 1978 by the International Organization for Standardization (ISO). Nowadays, many national and international quality standards for laboratory practice exist and quality laboratory practice has become the norm. In the case of full compliance with standard requirements a laboratory may be accredited: a formal recognition of competence and compliance to a quality standard.

Documents other than standards are important as well. Quality standards are lists of requirements that need to be met in order to ensure quality practice. These standards do not contain explanations on how to implement the requirements or comply with them. In addition, standards are mostly not measurable. Therefore, a laboratory needs guidelines describing how to implement the standard requirements and a checklist to visualise the degree to which it has met the standard.

Since 2008 the initiatives to strengthen laboratories in low and middle income countries (LMIC) have evolved rapidly, especially in the clinical diagnostic laboratory sector.^3^ Whilst efforts to increase health in LMIC initially focused on improving treatment and care, the focus has now broadened to also improving laboratory diagnosis. Stakeholders encourage countries to develop national standards, guidelines and regulations (hereafter called ‘quality documents’) to improve the quality of laboratory testing, contributing to meeting the Millennium Development Goals (MDG) to achieve better health.^4,5,6^ In 2009 the World Health Organization (WHO) regional office for Africa (WHO-AFRO) launched an accreditation checklist based on international quality documents^5,7,8,9,10,11^ and in 2005 the WHO regional office for South East Asia (WHO-SEARO) recommended the expansion of the national accreditation scheme of Thailand to member countries.^2,12^

The number of international laboratory quality documents is large and their scopes are diverse. As a result, deciding which document to use for implementing QM may be difficult: the authors have observed clinical laboratories implementing quality standards meant for non-clinical laboratories. This points to a lack of knowledge of decision makers regarding the existence of different types of quality documents for different types of laboratories. No review of widely used laboratory quality documents has been published to describe their characteristics and differences. We believe that there is a need for such information in the context of the rapidly increasing efforts to introduce QM in clinical laboratories. Here we review documents that are widely used in QM implementation in laboratories, followed by an analysis of selected documents.

The target audience of this study is the clinical laboratory sector. However, non-clinical laboratory quality documents were also included to provide perspective by showing their differences when compared with clinical laboratory quality documents (explaining why they should not be used by clinical laboratories). This analysis is intended for policy makers developing national laboratory quality policies, and for laboratory managers and staff with the ambition to implement a quality management system (QMS).

## Methodology

### Selection of laboratory quality documents

Laboratory QM development was reviewed using an unstructured Internet search to identify quality documents that are internationally important and widely used. Of these, seven quality documents were selected for further study – five international standards or guidelines and two national US regulations.

ISO 17025 – *General requirements for test and calibration laboratories* was included because it was the first internationally published standard (in 1978) on laboratory QM, originally named ISO Guide 25.^13^ This standard is presently widely used for testing and calibration laboratories.

In 1979 in the USA, the Food and Drug Administration (FDA) enacted a national regulation called *Good Laboratory Practice for Nonclinical Laboratory Studies* (21CFR58) (hereafter called FDA-GLP).^14,15^ In 1981 the Organization for Economic Cooperation and Development (OECD) translated the FDA-GLP into international requirements for testing and calibration laboratories in its member states, titled *Principles on Good Laboratory Practice* (OECD-GLP), consisting of a series of documents focusing on the different aspects of accreditation.^16^ The OECD-GLP could be regarded as the second quality document to ever be developed specifically for international use.

In 1988 a national regulation made expressly for clinical laboratories was enacted in the USA. This regulation is known as *Clinical Laboratory Improvement Amendments* (CLIA), coded 42CFR493.^17^ The CLIA is a national regulation, but the College of American Pathologists (CAP) uses it as the basis for its accreditation^18^ that is provided to clinical laboratories worldwide. Therefore, the CLIA is also of international significance.

ISO 15189 – *Medical Laboratories – Particular requirements for quality and competence* is the most widely used clinical laboratory standard. It was published for the first time in 2003.^19^ The version used in our analysis is from 2007.

In 1999 the Clinical and Laboratory Standards Institute (CLSI) developed GP26-A1, a guideline for establishing a QMS in clinical laboratories. The development of the guideline started in 1997 by combining all the requirements of six quality documents (current at that time) into one document.^20^ In 2006 the ISO 15189 requirements were incorporated into the third edition of this guideline (GP26-A3) titled: *Application of a Quality Management System Model for Laboratory Services; Approved Guideline*.^21^ In the spring of 2011 the fourth edition was published (GP26-A4). This guideline is used internationally to implement the requirements of ISO 15189 in clinical laboratories.

The Joint Commission International (JCI) *Accreditation standard for clinical laboratories* (second edition published in 2010) has a rather different background from the other quality standards included in this study. The JCI provides accreditation to hospitals. As part of this accreditation, the JCI developed the standard to include QM practices in hospital laboratories.^22^ Because JCI hospital accreditation is widespread, the significance of JCI laboratory accreditation may also increase (Table 1 provides an overview of the selected quality documents for analysis, including background information).

**TABLE 1:** Overview of analysed quality documents with their most important characteristics.

Name	Category^†^	National or international^‡^	Type of document^§^	Developed or maintained by^¶^	First publication^††^	Used publication or edition^‡‡^
ISO 17025 General requirements for the competence of testing and calibration laboratories	Non-clinical	International	International standard	ISO	1999 (Guide 25: 1978)	2005/2nd edition
Good Laboratory Practice for Non-clinical Laboratory Studies	Non-clinical	National (USA)	National regulation (USA)	FDA	1978	2009 (revision April 1)
Principles on Good Laboratory ractice	Non-clinical	International	International standard	OECD	1981	1998/1st revision
Clinical Laboratory Improvement Amendments	Clinical	National (USA)	National regulation (USA)	US Government	1988	2004 (revision January 24)
ISO 15189 Medical Laboratories – Particular requirements for quality and competence	Clinical	International	International standard	ISO	2003/1st edition	2007/2nd edition
CLSI GP26 Application of a quality management system model for laboratory service	Clinical	International	International guideline	CLSI	1999/A1	2004/A3
JCI Accreditation Standards for Clinical Laboratories	Clinical	International	International standard and guideline in one	JCI	2003/1st edition	2010/2nd edition

† The type of laboratory for which the quality document was developed: either clinical diagnostic laboratories or non-clinical testing or calibration laboratories.

‡ The focus area for use of the quality document: either national or international use.

§ The type of document: an international standard, an international guideline or a national regulation (regulatory standard), with the name of the country it was developed by between brackets.

¶ The organisation that developed the quality document.

†† The date the first edition was published.

‡‡ The date and edition used for analysis.

### Analysis of quality documents

To guide the analysis, a framework was constructed consisting of three steps to analyse the selected quality documents. In step 1 the nature of the quality document was determined. We used the following definitions:

- International standard: a consensus document developed for international use, summing up all the requirements for a QMS.
- International guideline: a more descriptive document than a standard, developed for international use, defining the intent of standard requirements and how these should be integrated in the laboratory processes.
- National regulation: regulatory standard written by a national government.

**FIGURE 1:**
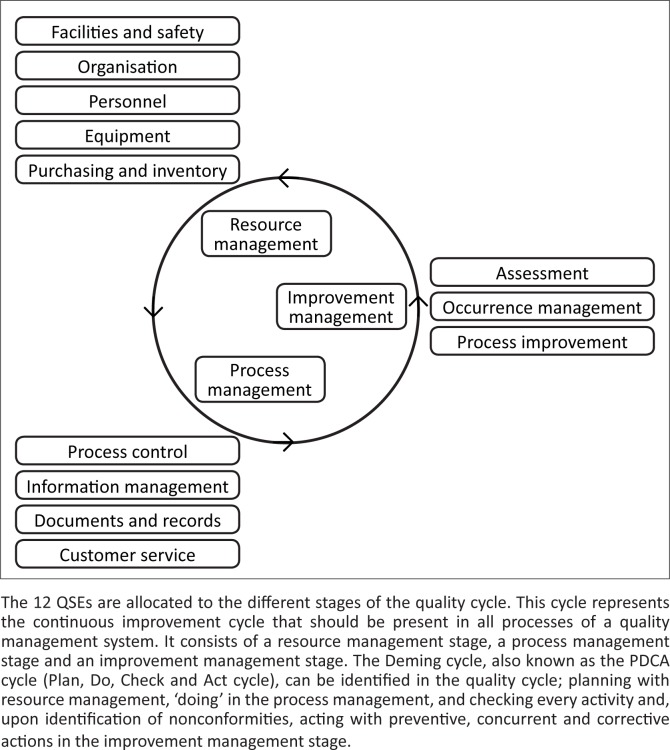
The framework with the 12 Quality System Essentials used to analyse to which extent the content of quality documents covers all aspects of total quality management.

In step 2 a framework of 12 Quality System Essentials (QSEs) was adapted from the CLSI guideline for implementing QM in clinical laboratories: GP26-A3.^21^ These 12 QSEs together cover all aspects of a QMS (Figure 1). When analysing the selected quality documents, their articles or sub-parts were allocated to one of the 12 QSEs to yield an indication of the level of coverage of total quality by each quality document (Table 2). Finally, in step 3 additional characteristics of each quality document were recorded.

**TABLE 2a:** Analysis of contents of national quality documents using the Quality System Essentials framework.

Quality System Element (QSE)	National quality documents	
	Non-clinical	Clinical
	FDA GLP for non-clinical testing	CLIA
Facilities and safety	Sub-part C: 58.41 / 58.43 / 58.45 / 58.47 / 58.49 / 58.51	Sub-part J: 493.1100-493.1101
Organisation	Sub-part B: 58.31 / 58.33 / 58.35	Sub-part K: 493.1200-.1239 / 493.1242-.1249 / 493.1282 / 493.1289 / 493.1299
Personnel	Sub-part B: 58.29	Sub-part M: 493.1351-.1495
Equipment	Sub-part D: 58.61 / 58.63	Sub-part K: 493.1252-.1255
Purchasing and inventory	X	Sub-part K: 493.1252
Process control	Sub-part E: 58.81 / 58.83 / 58.90 / Sub-part F: 58.105 / 58.113 / Sub-part G: 58.120 / 58.130a-d	Sub-part K: 493.1232 / 493.1240-.1242 / 493.1250-.1251 / 493.1256-.1282 / 493.1290
Information management	Sub-part G: 58.130e / Sub-part J: 58.185	Sub-part K: 493.1231 / 493.1291
Documents and records	Sub-part J: 58.190 / 58.195	Sub-part J: 493.1101e / 493.1105 / Sub-part K: 493.1283
Customer service	X	Sub-part K: 493.1233-.1234 / 493.1291e / Sub-part M: 493.1407 / 493.1419
Assessment	Sub-part K: 58.200 / 58.202 / 58.204 / 58.206 / 58.210 / 58.213 / 58.215 / 58.217 / 58.219	Sub-part H: 493.801-.807 / Sub-part K: 493.1239 / 493.1249 / 493.1253-.1254 / 493.1289 / 493.1299
Occurrence management	X	Sub-part K: 493.1233-.1234 / 493.1282
Process improvement	X	Sub-part K: 493.1236-.1239 / 493.1249 / 493.1289 / 493.1299

A dash (-) indicates a separation (e.g. chapter 1-3: chapters 1 till 3), a slash (/) indicates a sum (e.g. chapter 1 / 3: chapters 1 and 3).

X, indicates no contents of the quality document to the related QSE.

**TABLE 2b:** Analysis of contents of international quality documents using the Quality System Essentials framework.

Quality System Element (QSE)	International quality documents				
	Non-clinical		Clinical		
	OECD Principles on GLP	ISO 17025:2005	ISO 15189:2007	CLSI GP26-A3	JCI clinical laboratory standard
Facilities and safety	3	5.3	5.2 / B2	6.12	MGT.4.7-.4.9 / RSM.3-.3.2.1 / RSM.5-.7.3
Organisation	1.1.1 / 1.1.2a / 1.1.2e-q / 1.2 / 2.2	4.1 / 4.2 / 5.1	4.1 / 4.2	6.2	MGT.1-.1.1 / MGT.1.3-.2.2 / MGT.4-.4.1.3
Personnel	1.1.2b-c	5.2	5.1	6.3	RSM.1-.2
Equipment	4/5	5.5	5.3 / B1 / B7 / B8	6.4	RSM.1 / RSM.4-.4.3
Purchasing and inventory	(guidelines provided separately)	4.4 / 4.5 / 4.6	4.4 / 4.5 / 4.6	6.5	MGT.1.2-.1.2.2 / RSM.1 / RSM.4.4-.4.5
Process control	6 / 7 / 8.1.1-8.3.2	5.4 / 5.6 / 5.7 / 5.8 / 5.9	5.4 / 5.5 / 5.6 / 5.7 / 5.8 / C4 / C5 / C6 / C9	6.6	MGT.4.2-.4.6 / DCP.2-.5 / QCP.1-.14.6
Information management	8.3.3-8.3.5 / 9	5.10	5.8 / B4 / B5 / B6 / C3 / C4 / C6 / C8	6.7	X
Documents and records	10	4.3 / 4.13	4.3 / 4.13 / C7 / C8	6.1	DCP.1 / DCP.5
Customer service	X	4.2.4 / 4.7 / 4.8	4.7 / 4.8	6.11	MGT.3-.3.2
Assessment	(guidelines provided separately)	4.14 / 4.15	4.14 / 4.15 / 5.6.4	6.9	MGT.4.1.4 / MGT.4.10
Occurrence management	X	4.9 / 4.11 / 4.12	4.9 / 4.10 / 4.11	6.8	DCP.4.3 / QCP.1.7
Process improvement	X	4.10	4.12	6.10	MGT.4-.4.1.6 / MGT.4.7-.4.9

A dash (-) indicates a separation (e.g. chapter 1-3: chapters 1 till 3), a slash (/) indicates a sum (e.g. chapter 1 / 3: chapters 1 and 3).

X, indicates no contents of the quality document to the related QSE.

## Results

The results of the analysis of the nature of the quality documents (analysis step 1) are provided (Table 1). The FDA-GLP, developed for non-clinical research laboratories, and the CLIA, developed for clinical diagnostic laboratories, are both national regulatory standards. The quality documents developed for international use included the OECD-GLP, ISO 17025:2005, ISO 15189:2007, CLSI GP26-A3 and the JCI clinical laboratory standard, 2nd edition. The OECD-GLP and ISO 17025 were developed for use in non-clinical laboratories and the ISO 15189, CLSI GP26 and JCI clinical laboratory standard were developed for use in clinical laboratories.

The results of determining the level of coverage of total quality by each quality document (analysis step 2) are provided (Table 2).

### Clinical quality documents

In general, customers play an important role in clinical laboratory quality documents. Whereas the non-clinical quality documents are generally written from the perspective of protecting the process and its product, the clinical quality documents are written to protect the customer from flawed results.

The attention given to continuous improvement is much higher in clinical quality documents than in non-clinical quality documents. Also, occurrence management (correct handling of nonconformities/accidents, followed by improvement measures to prevent similar occurrences in the future) receives much more attention in clinical quality documents than in non-clinical quality documents.

### ISO 15189

Of all clinical laboratory quality documents studied, ISO 15189 is probably the most widely used standard worldwide. Regional and international accreditation organisations such as the International Laboratory Accreditation Cooperation (ILAC), the InterAmerican Accreditation Cooperation (IAAC), the Asia Pacific Laboratory Accreditation Cooperation (APLAC) and the European Cooperation for Accreditation (EA) recommend accreditation of clinical laboratories to ISO 15189.^23^

A notable characteristic of the ISO 15189 standard is, besides the requirements to a QMS, the inclusion of two annexes with recommendations: one contains all recommendations for a laboratory information system and another is completely dedicated to different aspects of ethics.^24^

### CLSI GP26

As a standard, ISO 15189 is a document that only contains requirements for a QMS, but no further explanation on why and how these requirements should be complied with. The CLSI GP26 guideline contains much more information for laboratories on what QM is, how it is integrated in the laboratory work, and why certain activities should be performed. The CLSI GP26 approaches every laboratory activity from a process workflow perspective. It contains a detailed description of this workflow by discussing each phase of the process using flow charts and process tables. The remainder of the document describes the requirements per QSE, which are highly focused on continuous improvement (when compared to other clinical laboratory quality documents).

### The Clinical Laboratory Improvement Amendments

The CLIA is a US federal document that applies to all clinical laboratory testing performed on humans except for clinical trials and fundamental research. This regulation is comprehensive and contains many discipline specific requirements (i.e. special requirements for histopathology, genetic testing, molecular techniques, etc.). Remarkably, the nature of the CLIA is more similar to non-clinical quality documents as it is primarily focused on protecting the analysis process rather than customers and personnel. However, the CLIA, in contrast to most non-clinical standards, covers all 12 QSEs, which indicates that it is specifically designed for clinical laboratory practice.

### JCI Clinical Laboratory Standard

The JCI accreditation standard is slightly different from the aforementioned standards as it is a highly elaborate document that combines a standard with a guideline, describing the intent of each standard requirement and, uniquely, the measurable elements of each requirement. It is most elaborate on safety requirements, but it is the only clinical laboratory document which does not cover all 12 QSEs: information management is not covered as a topic in itself, although some requirements related to information management are present as part of different sections in the document. This standard is, similarly to the CLIA, highly elaborate on sub disciplines within laboratory practice providing quality assurance and quality control standards for each specific discipline.

### Non-clinical quality documents

The most salient findings are that the non-clinical quality documents are less customer focused and, instead, written from the perspective of protecting the process and its product.

Non-clinical quality documents are also less focused on continuous improvement; both the FDA-GLP and OECD-GLP lack requirement on this QSE. In addition, the FDA-GLP also lacks requirements on the purchasing and inventory element of the QMS.

Another salient characteristic of most non-clinical quality documents (FDA-GLP, OECD-GLP, and ISO 17025) is the requirement to have a study plan or validated protocol in place besides Standard Operating Procedures (SOPs).

## Discussion

There are a number of quality documents specific for clinical laboratories and for non-clinical laboratories. We compared the characteristics, suitability and applicability of these quality documents in view of the increasing efforts to introduce QM in laboratories. Clinical quality documents focus on both protecting the process, and the safety of the customer and the laboratory staff, whereas non-clinical documents aim primarily at protecting the safety and integrity of the analyses performed. Moreover, non-clinical quality documents are generally not focused on continuous improvement, an aspect that could be regarded as one of the major goals of a QMS.

### Clinical laboratory quality documents

The fact that ISO 15189 includes a complete annex on ethics is exceptional when compared to all other quality documents. In clinical laboratory practice patient confidentiality and proper behaviour towards the patient are obvious ethical requirements. However, regulations regarding financial arrangements of the laboratory staff with external organisations or persons, or protection of the environment through correct waste-management are also ethical requirements. Therefore, we recommend including a paragraph containing specific ethical norms with regard to laboratory practice in every quality document.

It was observed that the CLIA is highly comprehensive when compared to other clinical quality documents. This may be related to the fact that the CLIA is adapted to a national situation (with laboratories in the USA generally having abundant resources to facilitate good quality practice), whilst ISO 15189, as an international standard, has to maintain a certain level of generality to make it applicable in multiple countries that may have different standards of practice that are often determined by resource availability.

The CLIA is more focused on protecting the process rather than the customers and personnel, making it different compared to other clinical quality documents. This may be a typical characteristic of a regulation that can have a narrower focus because other national regulations cover, for example, occupational safety (e.g. the USA 29 CFR part 1910 ^25^): clinical laboratories in the USA following the CLIA need to comply with multiple other national regulations, whereas international standards and guidelines have to provide complete sets of requirements covering all aspects of laboratory practice.

### Non-clinical laboratory quality documents

Most non-clinical laboratory quality documents are primarily process-focused. This may be the reason for the total absence of personnel safety requirements: the paragraphs of non-clinical quality documents shown in Table 2 in the QSE *Facilities and Safety* are all related to proper facilities and actions enabling safety of the process, not directed at the safety of personnel. This is illustrated by the requirement of the FDA-GLP in sub-part B, 58.29 (d): ‘personnel shall take necessary personal sanitation and health precautions designed to avoid contamination of test and control articles and test systems’.

A characteristic found to be specific for non-clinical quality documents is the requirement to have a study plan or validated protocol in place besides SOPs. Often, non-clinical laboratories perform research in addition to routine procedures. This research cannot be standardised in SOPs.

Notable, and probably related to the nature of test and calibration laboratories, is the low number of requirements aiming at the QSE *Process Improvement*. In clinical laboratories, processes consist mainly of routinely performed procedures. Continuous improvement is necessary to keep the performance of these procedures as efficient and effective as possible. In calibration laboratories, techniques should be performed with as little variance as possible, whereas variation in activities is inherent to the work of testing laboratories. Incorporating continuous improvement is therefore complicated, if not impossible.

QSEs that are left uncovered in all but one of the non-clinical quality documents are *Occurrence Management* and *Customer Service*. Establishing procedures on customer service and occurrence management may be advisable in any environment. Only ISO 17025 contains requirements related to both QSEs, probably due to the incorporation of the highly customer-focused ISO 9001 standard during its development.^13, 26^

### National versus international laboratory quality documents

In many countries, international quality documents serve as the basis for national quality documents. Such adaption leads to documents that vary by country with regard to comprehensiveness. For example, the national guideline for clinical laboratories in the Netherlands is more extensive than the ISO 15189 standard.^27^ The same applies to the USA CLIA and FDA-GLP regulations. In contrast, the Chinese standard is less extensive than the ISO 15189, which makes it more feasible for laboratories in China to attain the standard.^28^ The required level of ISO 15189 was considered to be too high in relation to the resources available, with the consequence that few laboratories in China tried to become accredited. By making the national standards easier to achieve, Chinese laboratories were encouraged to implement QM leading to a substantial increase in accredited laboratories.^28^ On one hand some efforts on QM are better than no efforts at all, the concern however is the question in how far such simplified national standards can maintain adequate quality. This should be a topic of further research.

Nevertheless, it is recommended to use national quality regulations, if available, as they are often more detailed and optimally adapted to the national situation, and take into account the available resources. The problem is that national standards are currently almost exclusively available in high-income countries. In the last decade, several middle-income countries, for example Thailand, Mexico and Argentina, have developed simplified national accreditation schemes based on international standards.^29,30,31,32^ Recently, WHO-AFRO together with, among others, the USA Centers for Disease Control and Prevention (CDC) has launched an accreditation checklist based on CLSI GP26 and ISO 15189, which is tailor-made for implementation in clinical laboratories in LMIC, and has started the roll-out in sub-Saharan Africa.^7^ A strong point is that the absence of national regulations is taken into account in this initiative: the WHO-AFRO accreditation checklist contains lots of questions regarding laboratory safety that would otherwise need to be covered by national regulations on occupational safety.^11^ ISO 15189 only refers to national or regional regulations for safety requirements in such instances; these are absent in many countries.

### Study limitations

A potential weakness of this study is that the framework for analysis was adapted from a clinical laboratory quality document. This means that this framework was tailor-made for clinical laboratory quality documents and thus biased towards a clinical laboratory QMS. Although we therefore were able to correctly identify gaps in non-clinical quality documents compared to clinical quality documents, we were not able to identify gaps in clinical laboratory quality documents as compared to non-clinical quality documents.

### Recommendations

The type of laboratory and the resources available determine which document suits the practice of the laboratory best. National regulations are generally more detailed and tailor-made to the national laboratory system. International guidelines may be less detailed in order to remain applicable in multiple countries.

Laboratories planning to establish a QMS need both a standard and a guideline. A standard provides no information on the reasons for implementation of the requirements, and standards are generally not measurable. The JCI document illustrates the need for more than a standard: in this document the requirements (standard) are supplemented with a description of the intent of the requirements to prevent misinterpretation (guideline). In addition, measurable elements help the laboratory to determine whether each requirement is complied with.

It is important that documents are chosen which suit the practice of a laboratory best. Hence, clinical laboratories should choose a clinical laboratory standard, not the FDA-GLP, OECD-GLP, ISO 17025 or any other non-clinical laboratory document.

Several suggestions and recommendations remain to developers of quality documents. It was observed that non-clinical quality documents generally lack provisions on occurrence management, customer service and process improvement; we recommend including requirements on these aspects. Ethics is also an element that applies to all types of organisations. Increasing attention to ethical behaviour is highly recommended.
